# Designing high-efficiency extended depth-of-focus metalens via topology-shape optimization

**DOI:** 10.1515/nanoph-2022-0183

**Published:** 2022-05-13

**Authors:** Yuhan Zheng, Mingfeng Xu, Mingbo Pu, Fei Zhang, Di Sang, Yinghui Guo, Xiong Li, Xiaoliang Ma, Xiangang Luo

**Affiliations:** State Key Laboratory of Optical Technologies on Nano-Fabrication and Micro-Engineering, Institute of Optics and Electronics, Chinese Academy of Sciences, Chengdu 610209, China; School of Optoelectronics, University of Chinese Academy of Sciences, Beijing 100049, China; Research Center on Vector Optical Fields, Institute of Optics and Electronics, Chinese Academy of Sciences, Chengdu 610209, China; College of Electronic Science and Technology, National University of Defense Technology, Changsha 410072, China

**Keywords:** extended depth-of-focus, metalens, topology optimization

## Abstract

Longitudinal optical field modulation is of critical importance in a wide range of applications, including optical imaging, spectroscopy, and optical manipulation. However, it remains a considerable challenge to realize a uniformly distributed light field with extended depth-of-focus. Here, a high-efficiency extended depth-of-focus metalens is proposed by adjoint-based topology-shape optimization approach, wherein the theoretical electric field intensity corresponding to a variable focal-length phase is utilized as the figure of merit. Using a dozen of metalens with random structure parameters as initial structures, the average focal depth of topology-shape optimized metalens is greatly improved up to 18.80 μm (about 29.7*λ*), which is 1.54 times higher than the diffraction-limited focal depth. Moreover, all the topology-shape optimized metalens exhibit high diffraction efficiency exceeding 0.7 over the whole focal depth range, which is approximately three times greater than that of the forward design. Our results offer a new insight into the design of extended depth-of-focus metalens and may find potential applications in imaging, holography, and optical fabrication.

## 1 Introduction

Over the past decades, the longitudinal optical field has attracted considerable attentions due to its extraordinary capability for optical manipulation. For example, using the focusing of radially polarized light to enhance the longitudinal field component can yield longitudinally polarized light needles [1, 2], which have potential applications in particle acceleration [3, 4] and particle capture [5]. Strongly focused longitudinal electric fields can also be obtained by the plasmonic lens combined with metallic tips [6, 7], which makes a significant contribution to tip-enhanced Raman spectroscopy [8] and near-field microscopy [9]. In addition, supercritical lens [10, 11] and multilevel diffractive lens [12, 13] are alternative approaches to increase the depth of focus, as well as reduce the cost and complexity of imaging systems.

Recently, subwavelength metasurfaces has garnered much interest due to its incredible capability to arbitrarily manipulate the amplitude, phase, and/or polarization of incident light [14], [15], [16], leading to promising applications in metalens [17], [18], [19], [20], [21], [22], meta-antennas [23], holography [24], [25], [26], etc. In comparison with traditional approaches, metalens is an excellent candidate to manipulate longitudinal optical field with extended depth-of-focus (DOF), which could be designed by using geometric phase metasurfaces [27] or varying the polarization of the incident light [28]. However, most of the previous works focused mainly on the modulation of the longitudinal field while suffering from the low efficiency, thus impeding their practical applications. It is a great challenge to design an extended DOF metalens with high efficiency over the entire DOF range.

From the perspective of design of metasurfaces, traditional methods are based on forward design through experience or parameters scanning to achieve the desired design goal. In contrast, topology optimization is an inverse design approach that utilizes the reciprocity of Green’s functions to perform electromagnetic adjoint simulation, leading to the high computation-efficiency of optimization process [29], [30], [31], [32]. Recently, topology optimization has been widely used to design metasurfaces for various applications, including large-angle metagrating [33], high-efficiency deflection metasurface [34], and disordered metasurface [35]. However, the finally optimized topological shapes of such freeform metasurfaces are not controllable, resulting in an inevitable difficulty for practical fabrication [36]. By contrast, topology-shape optimization only modifies the boundary position of unit structure while keeping the topology shape unchanged during the optimization process, which is more compatible with practical fabrication [37]. Recently, a broadband metasurface with an extended DOF has been demonstrated [38]. However, its efficiency is slightly reduced compared to that of conventional metalens.

**Figure 1:**
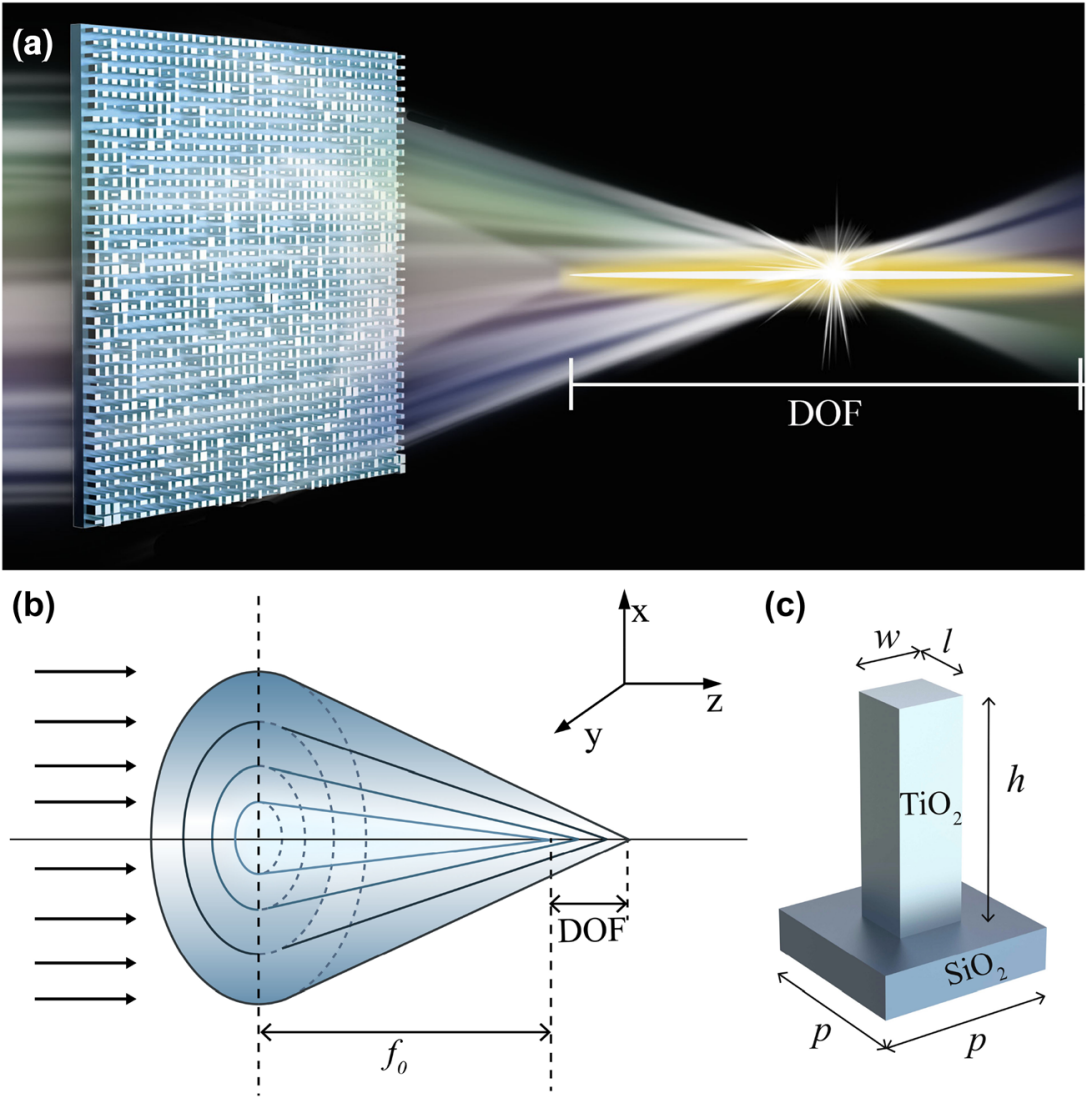
High-efficiency extended DOF metalens. (a) Schematic diagram of the metalens. (b) Focused optical path of the metalens with a variable focal-length phase. (c) Unit structure of metalens.

Here, we inversely design a high-efficiency extended DOF metalens via topology-shape optimization approach, as shown in Figure 1a. Specifically, a variable focal-length phase [39, 40] is adopted as the theoretical phase distribution, and the figure of merit (FoM) is defined as the electric field intensity corresponding to the theoretical phase. Based on the initial random configuration, we inversely optimized 10 different sets of structures, all of which exhibit extended DOF with high focusing efficiency. As a result, the optimized metalens has a DOF of 18.80 μm on average (about 29.7*λ*), which is 1.54 times higher than the diffraction-limited focal depth (about 19.29*λ*). In addition, the average focusing efficiency of the metalens is optimized to 72.57%, which is more than 30 times higher than that of initial random structure. Moreover, the topology-optimized metalens has a high resolution of 1.75 μm, which is basically below the diffraction limit. Our results provide a useful methodology for ultra-long focal depth lens design and the proposed high-efficiency extended DOF metalens may find potential applications in optical imaging, holography, and lithography.

## 2 Principle of topology-shape optimization

### 2.1 Variable focal-length phase

To obtain an extended DOF while maintaining high efficiency, a variable focal-length phase is adopted as the theoretical phase distribution. As shown in Figure 1b, this phase mimics a unique optical element, the so-called axicon, which could overcome the constraints between extended DOF and high resolution to generate an almost nondiffracting beam. Since axicon can only converge very low energy into the focused beam, this phase also exhibits the high focusing feature of spherical phase to improve the focusing efficiency. Under the paraxial approximation condition, the focusing spherical phase of an ordinary lens is given by ref. [39].

(1) $$\phi \left(r\right)=\frac{2\pi }{\lambda }\cdot \frac{r^{2}}{2f}\text{,}$$

where *r* is the radial coordinate (0 < *r* < *R* and *R* is the radius of the lens) and *f* is the focal length (see S1, Supporting Information for details). When the light rays converge away from the lens surface, the focal length *f* can be written as [39].

(2) $$f\left(r\right)=f_{0}+ar^{b}\text{,}$$

where *f*_0_ is the starting position of the focus depth of the lens. *a* and *b* are the constant parameters that determines the distribution of rays.

To obtain a uniform DOF, the intensity distribution parameters are determined as *b* = 2, leading to *a* = DOF/*R*^2^ (see S1, Supporting Information for details). Here, the DOF is defined as the position where the electric field intensity along the *z*-axis is greater than 80% of the maximum. Therefore, the theoretical phase of target metalens with a variable focal-length is expressed as

(3) $$\varphi_{\text{ideal}}\left(r\right)=\frac{\pi }{\lambda }\cdot \frac{r^{2}}{f_{0}+\frac{\text{DOF}}{R^{2}}\cdot r^{2}}\text{.}$$

In the simulations of topology-shape optimization, the initial focal length is set to 30 μm and the DOF is 20 μm, which means that the focal point is located at 40 μm. The radius *R* is set to 10 μm, corresponding to a maximum *NA* = 0.33 at the wavelength *λ* = 632.8 nm in the TM-polarization. As the computational time increases significantly with the increment of metalens size, a moderate radius of 10 μm is chosen based on the consideration of the balance between the computation efficiency and the metalens size. As shown in Figure 1c, the substrate material is SiO_2_ and the unit structure is composed of TiO_2_ nanorods with varying dimensions (100–400 nm). Moreover, the height and the lattice period are 600 nm and 500 nm, respectively.

### 2.2 Topology-shape optimization

In the topology-shape optimization, the FoM is defined as the electric field intensity of the theoretical variable focal-length lens as mentioned above:

(4) $$\text{FoM}={\left|E\right|}^{2}={\left|E_{0}\cdot \;\exp\left(i\varphi_{\text{ideal}}\right)\right|}^{2}={\left|E_{0}\cdot \;\exp\left(i\frac{\pi }{\lambda }\cdot \frac{r^{2}}{f_{0}+\frac{\text{DOF}}{R^{2}}\cdot r^{2}}\right)\right|}^{2}\text{,}$$

where *E*_0_ is the amplitude of the electric field.

The adjoint source specified by the FoM during the adjoint simulation is governed by

(5) $$E_{\text{adj}}=G\left(x,x^{\prime }\right)\cdot \frac{\partial \text{FOM}}{\partial E}=G\left(x,x^{\prime }\right)\cdot E^{*}\text{,}$$

where *G*(*x*,*x*′) is the Green’s function in the sense that the electric dipole at *x*′ excites the electric field at point *x*, and ^∗^ denotes the complex conjugation. Using the above adjoint source, the boundary variation of the metalens could be determined in every iteration of topology-shape optimization (See S2, Supporting Information for details).

## 3 Results and discussion

**Figure 2:**
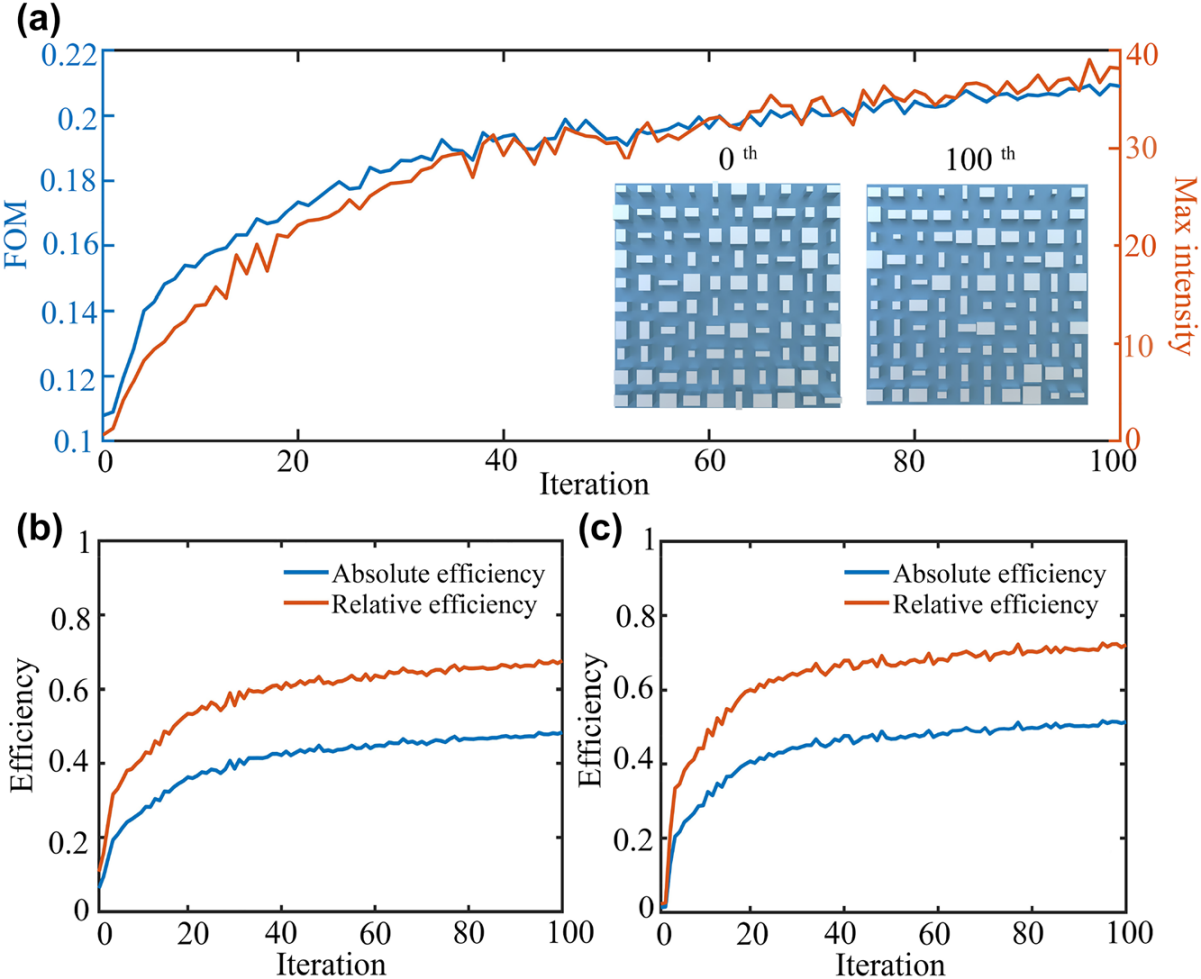
Topology optimization process of high-efficiency extended DOF metalens. (a) FoM and maximum intensity evolution for theoretical focus. The insets show the central 5 µm × 5 µm geometric layout of the random initial (0th iteration) and optimized structures (100th iteration). (b and c) Efficiency evolution for theoretical focus and actual focus.

The topology-shape optimization of high-efficiency extended DOF metalens is demonstrated based on 10 initial random structures. Figure 2 presents the optimization process and result for one of typical structures. As shown in Figure 2a, after 100 iterations of topology-shape optimization, the FoM raises from 0.11 to 0.21 while the maximum electric field intensity at the theoretical focal point increases from 0.60 to 38.11. Note that the undulations of FoM curve are caused by using a marginally larger step size, which can increase the search space without failing to find the optimal solution and getting trapped in a local optimum. In fact, each iteration always moves towards the gradient direction and tunes back in time when it deviates from the optimal path due to the slightly larger step size. Particularly, the geometric shape of unit structure keeps unchanged during the optimization process, while the corresponding dimension is updated to meet the improvement of FoM. Especially, the lattice centers of unit structure exhibit a slight movement, as shown in the insets of Figure 2a.

Figure 2b presents the improvement of absolute and relative efficiency at the theoretical focal points (i.e., the point at 40 μm away from the metalens). Here, the absolute efficiency is defined as the energy ratio of three times of the theoretical full width at half-maxima (FWHM) to the total incident energy, and the relative efficiency is defined as three times of the theoretical FWHM compared to the total power on the theoretical focal plane. In particular, the absolute efficiency increases from 6.43 to 48.29% (about 7.51 times), and the relative efficiency rises from 10.76 to 67.65% (about 6.28 times). Moreover, as shown in Figure 2c, the absolute efficiency of the actual focus has been improved from 1.41 to 51.46% (about 36.50 times), and the diffraction efficiency increased from 2.35 to 72.09% (about 30.68 times). It is worth noting that the definition of efficiency is almost the same as that of the theoretical focus, with exception that the theoretical FWHM is replaced by the FWHM of the actual focal plane.

**Figure 3:**
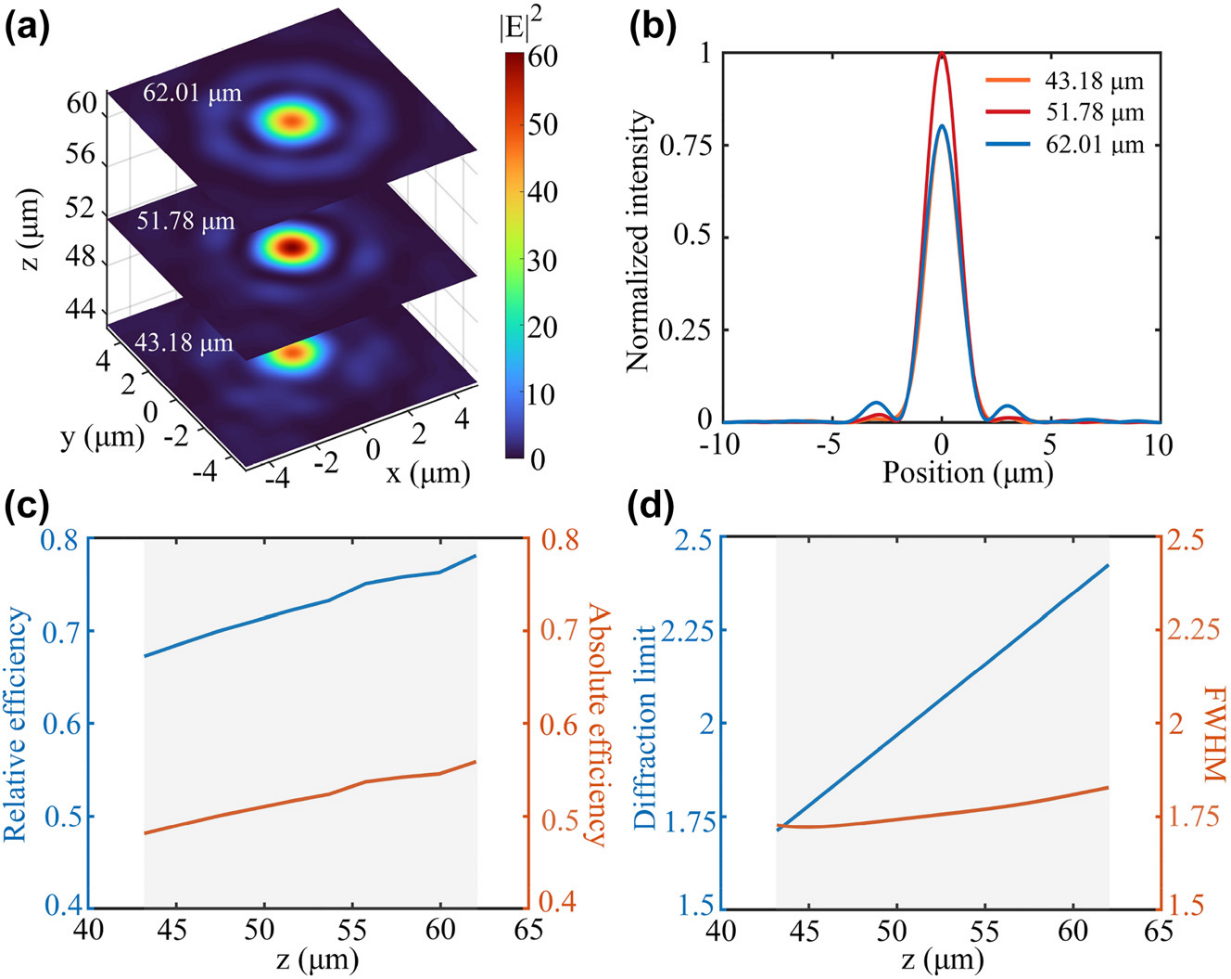
Optical field distribution over DOF range. (a) Electric field distribution in *xoy* plane at different locations in DOF (minimum, focal point, and maximum). (b) Normalized intensity distribution along the *x*-direction in DOF (minimum, focal point, and maximum). (c) Efficiency variation during DOF. (d) Diffraction limit and FWHM variations during DOF. The gray areas in (c) and (d) indicate the range of DOF.

Then we analyze the optical field distribution over the DOF range. As shown in Figure 3a and b, the optical field focuses effectively in the *xoy* plane at three different locations (minimum, focal point, and maximum) along the DOF direction. Obviously, the maximum intensity appears at the focal point. The corresponding FWHMs are 1.68 μm, 1.72 μm, and 1.80 μm, respectively. As presented in Figure 3c, the mean value of diffraction efficiency in the DOF range is 72.76% and the mean value of absolute efficiency is 52.04%, demonstrating the high efficiency over the entire DOF range. Moreover, as shown in Figure 3d, the mean value of FWHW in DOF is vastly improved to 1.76 μm, leading to a high resolution below the diffraction limit.

**Figure 4:**
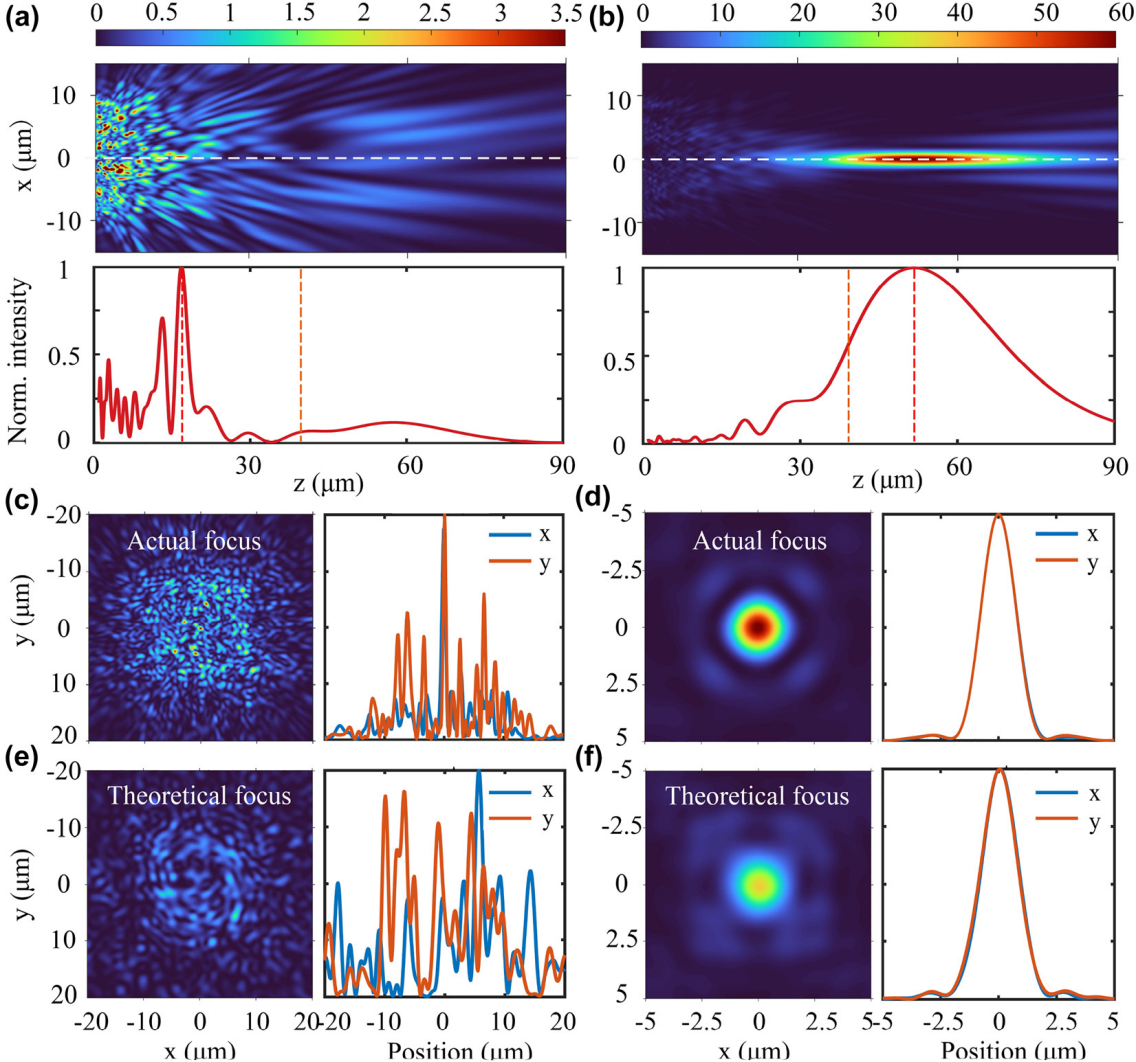
Intensity distributions for the initial random (left columns) and topology-shape optimized (right columns) structures. (a and b) intensity distributions along the *z*-direction. The orange and red dashed lines indicate the positions of the theoretical and actual focal points, respectively. The white dashed line indicates the cross-section of normalized intensity. (c–f) intensity distributions at the actual and theoretical focal point.

To verify the change of optical field caused by topology-shape optimization, we further investigate the optical field distribution at the theoretical focal point (i.e., *z* = 40 μm) and the actual focal point. As shown in Figure 4a and b, the optimized DOF extends from the initial 1.39–18.81 μm, which is close to theoretical designed value of 20 μm. Particularly, the topology-shape optimization improves the DOF at the theoretical maximum *NA* by a factor of 1.51, where the diffraction-limited DOF is defined as *λ*/*NA*^2^[41].

Figure 4c–f show that both the theoretical and actual focal points have been optimized from the dispersive spot to a focused state. In contrast to the spatially inhomogeneous distribution of the initial optical field, the optimized focal area exhibits a significantly symmetrical intensity distribution both along the *x* and *y* directions. Specifically, the FWHMs in the *x*-direction and *y*-direction at the theoretical focal point are 1.76 μm (2.78λ) and 1.80 μm (2.84λ), respectively. For the actual focal point, the corresponding FWHMs both in the *x*-direction and *y*-direction are 1.72 μm (2.72λ). Meanwhile, the actual focus is located at 51.78 μm, which has a particular offset compared to the theoretical location at 40 μm. On the one hand, such deviation results from the local optimization feature of topology-shape optimization, which is constrained by the limited iteration number and optimization parameter space. On the other hand, due to diffraction limitation, a smaller NA is more likely to realize extended DOF. Therefore, the focal point is automatically optimized toward the direction of decreasing NA. Note that offset of focal plane during topology-shape optimization process could be explained by the unequal interference effects along the *z*-axis [39]. Moreover, a possible solution to fix the focal length is to consider an additional limitation on the focal length position during the optimization process and then to perform a multi-objective topology optimization.

**Figure 5:**
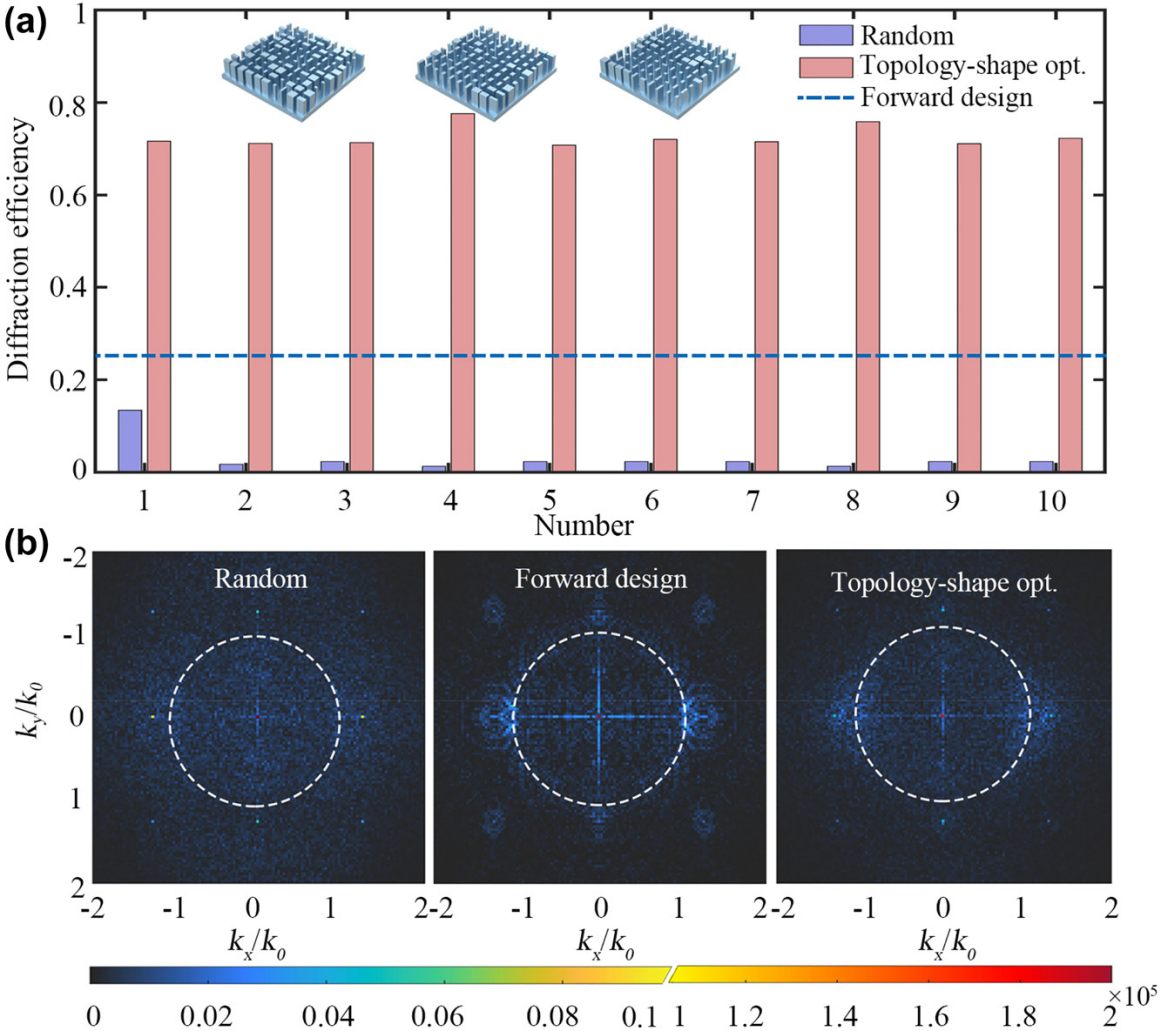
Optimized results for different initial random structures. (a) Diffraction efficiency comparison for initial random metalens, topology-shape optimized metalens, and forward-designed metalens. The insets show the structure of three typical optimized metalens at a 5 μm × 5 μm region. (b) Fourier transform of the electric field in the near-field (0.1*λ* from the structure) of *xoy* plane. From left to right are initial random metalens, forward-designed metalens, and topology-shape optimized metalens. The white dashed line corresponds to $k_{x}^{2}+k_{y}^{2}=k_{0}^{2}$ .

Finally, to demonstrate the generality of topology-shape optimization approach for the design of high-efficiency extended DOF metalens, the optimized results for 10 different sets of initial random structures are further investigated. As shown in Figure 5a, in comparison with the initial random structures, the average diffraction efficiency of optimized metalens is improved from 3.19% to 72.57% (about 22.75 times), indicating the validity of topology-shape optimization approach. Moreover, the optimized results show that the average DOF is optimized to 18.80 μm (about 29.7λ), corresponding to 1.54 times of the diffraction-limited focal length, with the FWHM being 1.75 μm below diffraction limit (See S4, Supporting Information for details). Furthermore, we also forwardly design the metalens based on the theoretical phase distribution (see S3, Supporting Information for details). Compared to the forward design efficiency of 25.37%, the efficiency of the topology-shape optimized metalens has nearly three times improvement, indicating the superiority of topology-shape optimization for high-efficiency extended DOF metalens design. Actually, a random configuration of initial structure allows the topology optimization to break the local limitation of design phase spaces, which is more likely to achieve better results compared with a predesigned structure [42].

In addition, Figure 5b shows the spatial spectrum in the near field of the initial random metalens, the forward-designed metalens, and the topology-shape optimized metalens. The spatial spectrum is obtained from the Fourier transform of the *xoy* plane field distribution at 0.1*λ* (i.e., 60 nm) from the emission plane of the metalens. In contrast with initial random structure exhibiting scattered irregular distribution in the Fourier space, the near field modes for both topology-shape optimized metalens and forward-designed metalens are more ordered and compact, especially for the low-order modes. Meanwhile, the proportion of higher-order modes (i.e., evanescent wave in the range of $k_{x}^{2}+k_{y}^{2}>k_{0}^{2}$ ) for the two designed metalens have almost the same density, i.e., 61.29% for topology-shape optimization and 61.64% for forward design. Particularly, the topology-shape optimized metalens has fewer higher-order modes compared to that of forward-designed metalens. It means that less energy will be lost in the near field, which allows that most of the energy to reach the focus point smoothly and explains why the topology-shape optimization approach enables a higher efficiency and a more extended DOF.

## 4 Conclusions

In summary, we proposed a high-efficiency metalens with extended DOF by using topology-shape optimization. In fact, by using the variable focal length phase as the optimization target, the DOF can be adjusted and designed arbitrarily according to different scenes. During the topology-shape optimization process, the offset of lattice center of each unit structure allows for a more accurate structure design and a higher efficiency. Meanwhile, topology-shape optimization approach does not modify the geometric shape of cell structures, offering a significant advantage compared to topology optimization. In addition, it is revealed that the smaller evanescent waves could facilitate the formation of the high-efficiency extended DOF metalens.

We have compared our work with other design methods for extended DOF metalens [11, 38, 43]. It is obviously seen that the superiority of the inverse design to achieve the extended DOF, where the DOF is much longer compared to the forward ones. More importantly, in comparison with other inverse design approaches, the topology-shape optimization enables a significant improvement of efficiency (see S5, Supporting Information for details). Moreover, the designed metalens could be fabricated with atomic layer deposition and electron beam lithography technique (see S6, Supporting Information for details).

As an inverse design method, topology optimization could break the shackles of conventional forward design paradigm where the meta-device performance is greatly depending on the researchers’ inherent experience and imagination. And compared with data-driven deep learning approach that has been recently demonstrated [44], topology optimization does not require a large number of training sets to achieve the construction of the structure, offering higher computation efficiency. In fact, the combination of topology optimization and deep learning approach is an exciting solution for the inverse design of large-scale meta-devices [45]. Furthermore, the topology-shape optimization approach can be extended to various applications of nanophotonics, opening a new avenue to the inverse design of exotic optical functionalities.

## Supplementary Material

Supplementary Material

## References

[1] Wang H., Shi L., Lukyanchuk B., Sheppard C., Chong C. T. (2008). Creation of a needle of longitudinally polarized light in vacuum using binary optics. *Nat. Photonics*.

[2] Yu A., Chen G., Zhang Z. (2016). Creation of sub-diffraction longitudinally polarized spot by focusing radially polarized light with binary phase lens. *Sci. Rep.*.

[3] Gupta D. N., Kant N., Kim D. E., Suk H. (2007). Electron acceleration to GeV energy by a radially polarized laser. *Phys. Lett. A.*.

[4] Cicchitelli L., Hora H., Postle R. (1990). Longitudinal field components for laser beams in vacuum. *Phys. Rev. A.*.

[5] Zhan Q. (2004). Trapping metallic Rayleigh particles with radial polarization. *Opt Express*.

[6] Zhang M., Wang J., Tian Q. (2013). Tip-enhanced Raman spectroscopy based on plasmonic lens excitation and experimental detection. *Opt Express*.

[7] Spektor G., David A., Gjonaj B., Bartal G., Orenstein M. (2015). Metafocusing by a metaspiral plasmonic lens. *Nano Lett.*.

[8] Anderson M. S. (2000). Locally enhanced Raman spectroscopy with an atomic force microscope. *Appl. Phys. Lett.*.

[9] Barwick B., Flannigan D., Zewail A. (2009). Photon-induced near-field electron microscopy. *Nature*.

[10] Qin F., Huang K., Wu J. F. (2017). A supercritical lens optical label-free microscopy: sub-diffraction resolution and ultra-long working distance. *Adv. Mater.*.

[11] Qin F., Liu B. Q., Zhu L. W. (2021). π-phase modulated monolayer supercritical lens. *Nat. Commun.*.

[12] Banerji S., Meem M., Majumder A., Sensale-Rodriguez B., Menon R. (2020). Extreme-depth-of-focus imaging with a flat lens. *Optica*.

[13] Mohammad N., Meem M., Shen B., Wang P., Menon R. (2018). Broadband imaging with one planar diffractive lens. *Sci. Rep.*.

[14] Pu M., Guo Y., Li X., Ma X., Luo X. (2018). Revisitation of extraordinary young’s interference: from catenary optical fields to spin−orbit interaction in metasurfaces. *ACS Photonics*.

[15] Kildishev A. V., Boltasseva A., Shalaev V. M. (2013). Planar photonics with metasurfaces. *Science*.

[16] Yu N., Capasso F. (2014). Flat optics with designer metasurfaces. *Nat. Mater.*.

[17] Chen W. T., Zhu A. Y., Sisler J., Bharwani Z., Capasso F. (2019). A broadband achromatic polarization-insensitive metalens consisting of anisotropic nanostructures. *Nat. Commun.*.

[18] Wang Y., Chen Q., Yang W. (2021). High-efficiency broadband achromatic metalens for near-IR biological imaging window. *Nat. Commun.*.

[19] Dou K., Xie X., Pu M. (2020). Off-axis multi-wavelength dispersion controlling metalens for multi-color imaging. *Opto-Electron Adv.*.

[20] Wang Y. L., Fan Q. B., Xu T. (2021). Design of high efficiency achromatic metalens with large operation bandwidth using bilayer architecture. *Opto-Electron Adv.*.

[21] Zhang S., Huo P. C., Wang Y. L. (2022). Generation of achromatic auto-focusing airy beam for visible light by an all-dielectric metasurface. *J. Appl. Phys.*.

[22] Pahlevaninezhad M., Huang Y. W., Pahlevani M. (2022). Metasurface-based bijective illumination collection imaging provides high-resolution tomography in three dimensions. *Nat. Photonics*.

[23] Sun S., He Q., Xiao S. (2012). Gradient-index meta-surfaces as a bridge linking propagating waves and surface waves. *Nat. Mater.*.

[24] Song Q., Baroni A., Sawant R. (2020). Ptychography retrieval of fully polarized holograms from geometric-phase metasurfaces. *Nat. Commun.*.

[25] Bao Y., Yan J., Yang X., Qiu C.-W., Li B. (2021). Point-source geometric metasurface holography. *Nano Lett.*.

[26] Gao H., Fan X., Xiong W., Hong M. (2021). Recent advances in optical dynamic meta-holography. *Opto-Electron Adv.*.

[27] Zhang Z., Yang Q., Gong M., Chen M., Long Z. (2020). Metasurface lens with angular modulation for extended depth of focus imaging. *Opt. Lett.*.

[28] Zhang Z., Wen D., Zhang C. (2018). Multifunctional light sword metasurface lens. *ACS Photonics*.

[29] Molesky S., Lin Z., Piggott A. Y. (2018). Inverse design in nanophotonics. *Nat. Photonics*.

[30] Jensen J. S., Sigmund O. (2011). Topology optimization for nano-photonics. *Laser Photon. Rev.*.

[31] Mansouree M., Kwon H., Arbabi E. (2020). Multifunctional 2.5D metastructures enabled by adjoint optimization. *Optica*.

[32] Hughes T. W., Minkov M., Williamson I. A. D., Fan S. (2018). Adjoint method and inverse design for nonlinear nanophotonic devices. *ACS Photonics*.

[33] Sell D., Yang J., Doshay S., Yang R., Fan J. A. (2017). Large-Angle, multifunctional metagratings based on freeform multimode geometries. *Nano Lett.*.

[34] Xu M., Pu M., Sang D. (2021). Topology-optimized catenary-like metasurface for wide-angle and high-efficiency deflection: from a discrete to continuous geometric phase. *Opt Express*.

[35] Xu M., He Q., Pu M. (2022). Emerging long-range order from a freeform disordered metasurface. *Adv. Mater.*.

[36] Su V.-C., Chu C. H., Sun G., Tsai D. P. (2018). Advances in optical metasurfaces: fabrication and applications. *Opt Express*.

[37] Miller O. D. (2012). *Photonic Design: From Fundamental Solar Cell Physics to Computational Inverse Design*.

[j_nanoph-2022-0183_ref_038] Bayati E., Pestourie R., Colburn S. (2021). Inverse designed extended depth of focus meta-optics for broadband imaging in the visible. *Nanophotonics*.

[39] Davidson N., Friesem A. A., Hasman E. (1991). Holographic axilens: high resolution and long focal depth. *Opt. Lett.*.

[40] McLeod J. H. (1954). The axicon: a new type of optical element. *J. Opt. Soc. Am.*.

[41] Born M., Wolf E. (1999). *Principle of Optics*.

[42] Yang J., Fan J. A. (2017). Topology-optimized metasurfaces: impact of initial geometric layout. *Opt. Lett.*.

[43] Bayati E., Pestourie R., Colburn S. (2020). Inverse designed metalenses with extended depth of focus. *ACS Photonics*.

[44] Luo Y., Zhao Y. F., Li J. X. (2022). Computational imaging without a computer: seeing through random diffusers at the speed of light. *eLight*.

[45] Jiang J., Fan J. A. (2019). Global optimization of dielectric metasurfaces using a physics-driven neural network. *Nano Lett.*.

