# Supplementary material for: Designing high-efficiency extended depth-of-focus metalens via topology-shape optimization
Source: Nanophotonics. 2022 May 13;11(12):2967–75. doi: 10.1515/nanoph-2022-0183 (PMC11501133; doi:10.1515/nanoph-2022-0183)
Supplement: Supplementary file 1 — Supplementary Material [file j_nanoph-2022-0183_suppl.docx]

Supporting Information

S1. Derivation of Equation (1) and the intensity distribution parameters

The focusing spherical phase of an ordinary lens is given by

. (S1)

Subject to the paraxial approximation condition ($f\gg r$), the corresponding Taylor expansion of Equation S1 results in the following expression:

, (S2)

where the constant term of $2\pi f/\lambda$ could be omitted, thus leading to the expression of the Equation (1).

Then, we deduce the intensity distribution parameters $a$ and $b$. As shown in Fig. 1b, the phases of different areas correspond to different focal points. To obtain a uniform DOF, each different focal points need to have a similar incident luminous flux:

$\delta f\left(r\right)=2\pi r\delta r\cdot A$, (S3)

where $A$ indicates a linear relationship between the focal length and the area of the lens. Integrating Equation (S3) and comparing it with Equation (2), it can be easily obtained $b=2$. Hence, the parameter $a$ can be described by the DOF when $b$ is substituted into Equation (2):

$a=\frac{DOF}{R^{2}}$. (S4)

S2. Boundary variation in topology-shape optimization

The gradient in each iteration is governed by two simulations in the forward and inverse directions [32]:

$\delta F=2\mathrm{Re}\int\delta x_{n}\left(\varepsilon_{2}-\varepsilon_{1}\right)E_{fwd}\cdot E_{adj}dS$, (S5)

where $\delta x_{n}$ is the deformation size at each point along the boundary in the normal direction, and $S$ is the entire surface area, with $\varepsilon_{1}$ and $\varepsilon_{2}$ representing external and internal material permittivity, respectively.

Therefore, according to Maxwell's boundary conditions, the changing electric field is expressed as

$E_{fwd}\cong E_{fwd\parallel}+\frac{D_{fwd\perp}}{\varepsilon_{2}}$, (S6)

where $E_{fwd\parallel}$ denotes the electric field component parallel to the boundary and$D_{fwd\perp}$ means the potential shift-vector perpendicular to the edge during the forward simulation.

Similarly, the adjoint electric field can be written as

$E_{adj}\cong E_{adj\parallel}+\frac{D_{adj\perp}}{\varepsilon_{1}}$. (S7)

where$E_{adj\parallel}$ and $D_{adj\perp}$ represent the electric field component and potential shift-vector parallel and perpendicular to the boundary during the adjoint simulation, respectively.

Substituting them into the Equation (S5), the final expression for the gradient is obtained as

$\delta F=2\mathrm{Re}\int\delta x_{n}\left[\left(\varepsilon_{2}-\varepsilon_{1}\right)E_{fwd\parallel}\cdot E_{adj\parallel}+\left(\frac{1}{\varepsilon_{1}}-\frac{1}{\varepsilon_{2}}\right)D_{fwd\perp}\cdot D_{adj\perp}\right]dS$. (S8)

Finally, the boundary variation of the metalens is govern by

$\delta x_{n}=2\mathrm{Re}\left[\left(\varepsilon_{2}-\varepsilon_{1}\right)E_{fwd\parallel}\cdot E_{adj\parallel}+\left(\frac{1}{\varepsilon_{1}}-\frac{1}{\varepsilon_{2}}\right)D_{fwd\perp}\cdot D_{adj\perp}\right]$. (S9)

S3. Forward-designed metalens with the theoretical phase

To compare with the topology-shape optimized metalens, we also directly design the metalens using the theoretical phase distribution. The forward design uses an eighth-order phase distribution and the FDTD simulation software is used for the selection of structural parameters. The structure of the metalens obtained from the forward design is shown in Fig. S1, which consists of eight different groups of cell structures. The dimensions of the unit structure are shown in Table S1.


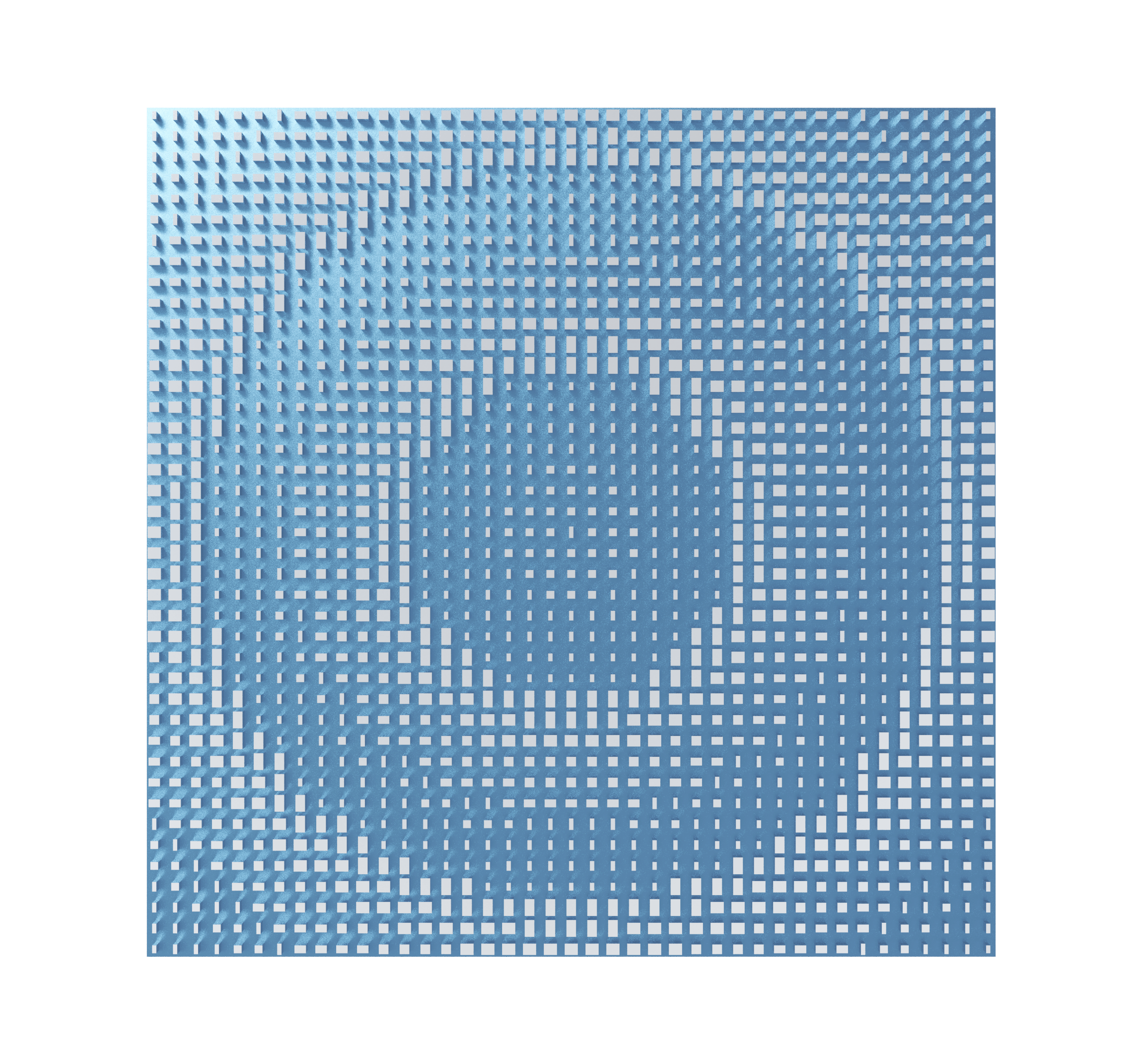


Figure **S1**: Structure of the forward-designed metalens.

Table S1: Cell structure dimensions for forward-simulated extended-depth-of-focus metalens

| Group | Length(nm) | Width(nm) | Phase | Group | Length(nm) | Width(nm) | Phase |
| --- | --- | --- | --- | --- | --- | --- | --- |
| 1 | 100 | 271.43 | $-\pi$ | 5 | 228.57 | 400 | $\frac{1}{4}\pi$ |
| 2 | 271.43 | 185.71 | $-\frac{3}{4}\pi$ | 6 | 100 | 185.71 | $\frac{1}{2}\pi$ |
| 3 | 228.57 | 228.57 | $-\frac{1}{2}\pi$ | 7 | 100 | 228.57 | $\frac{3}{4}\pi$ |
| 4 | 314.29 | 271.43 | $-\frac{1}{4}\pi$ | 8 | 185.71 | 185.71 | $\pi$ |


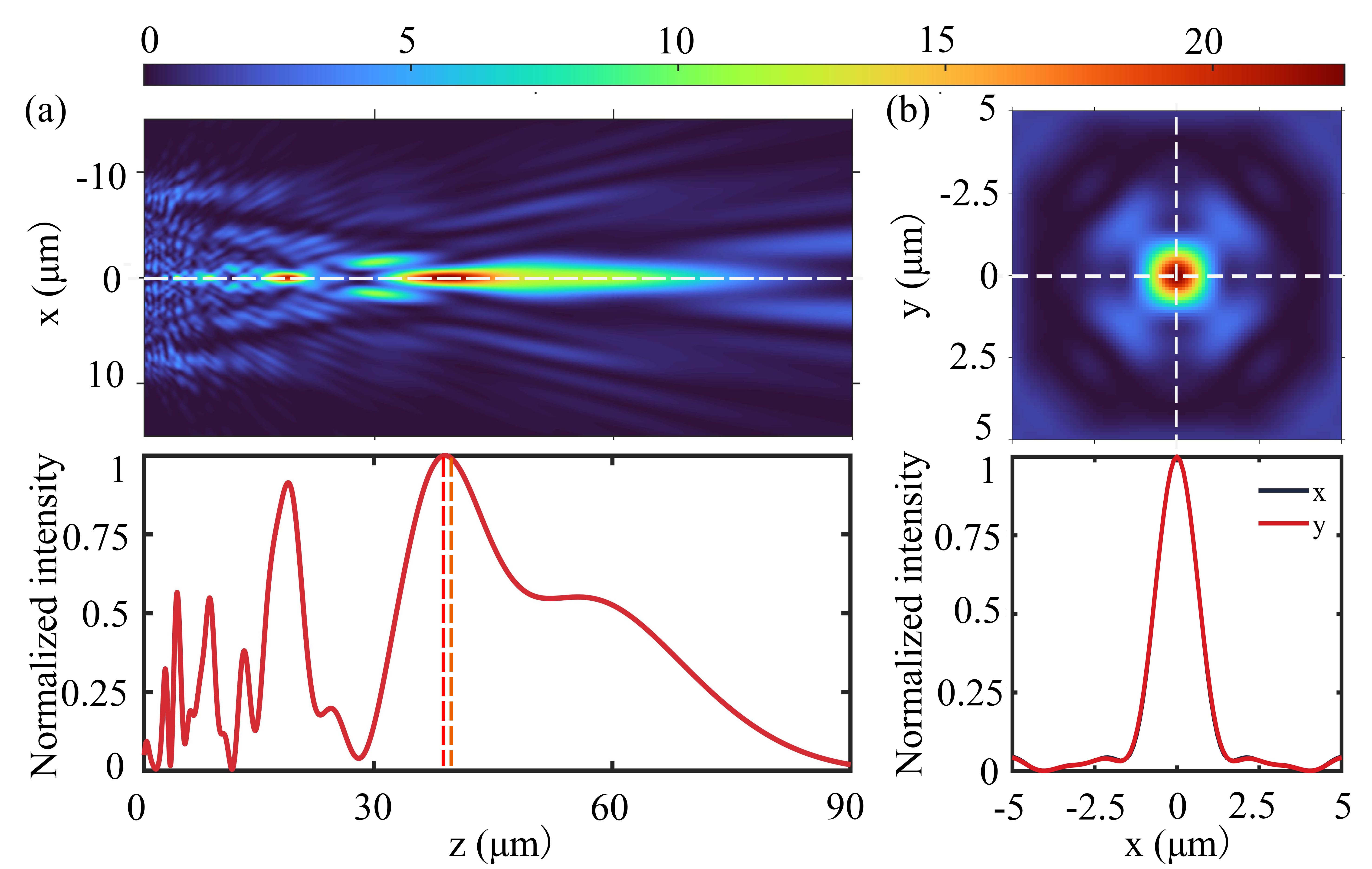


Figure **S2**: Simulation results of the forward-designed metalens. (a) Intensity distributions in the *xoz* plane. (b) Intensity distributions along the *z*-axis. The orange and red dashed lines indicate the positions of the theoretical and actual focal points, respectively. The white dashed line indicates the cross-section of normalized intensity.

As shown in Fig. S2, the focal point of this metalens is 38.93 μm, which is slightly deviated from the theoretical focal length of 40 μm. And the focal depth is 8.32μm, which is less than half of the theoretical focal depth. This can be explained by the fact that the parameters scanned during the forward simulation do not precisely match the theoretical phase. Especially, in the case of relatively large NA (for example, $NA_{\max}=0.33$), it is more difficult to obtain a better result. In addition, the absolute efficiency of such metalens is 17.92%, and the relative efficiency is 25.37%, which is about one-third of that of the topology-shape optimized ones. Moreover, the corresponding FWHM of forward-designed metalens is 1.40 μm, which is lower than the diffraction limit of 1.55 μm and is consistent with the high resolution of this theoretical phase. By comparing the results of forward and inverse design, the appeal of inverse design can be well illustrated.

S4. Optimization results for ten sets of initial random structures

Table S**2**: Ten sets of metalens parameters optimized by different initial random structures

| **Index** | **Focal length (μm)** | **Diffraction efficiency** | **FWHM (μm)** | **DOF improvement** |
| --- | --- | --- | --- | --- |
| 1 | 17.19 | 71.67% | 1.74 | 1.34 |
| 2 | 21.02 | 71.22% | 1.76 | 1.91 |
| 3 | 19.18 | 71.38% | 1.74 | 1.65 |
| 4 | 18.73 | 77.59% | 1.76 | 1.56 |
| 5 | 18.38 | 70.85% | 1.72 | 1.48 |
| 6 | 18.81 | 72.09% | 1.72 | 1.51 |
| 7 | 18.38 | 71.59% | 1.72 | 1.51 |
| 8 | 17.99 | 75.83% | 1.74 | 1.43 |
| 9 | 19.48 | 71.14% | 1.80 | 1.56 |
| 10 | 18.88 | 72.31% | 1.80 | 1.49 |

S5. Comparison of extended DOF metalens design

Table S**3**: Comparison of extended DOF metalens design

| **Reference** | **Design Method** | **Operating Wavelength** | **Side length /**  **Radius (μm)** | **NA** | **FWHM (μm)** | **DOF (μm)** | **Efficiency** |
| --- | --- | --- | --- | --- | --- | --- | --- |
| [11] | Forward design | 535nm | 40 | 0.4 | - | ≈10 | 1.2% |
| [38] | Inverse design | 410-700 nm | 500 | 0.44 | 24.9 | 400 | 47% |
| [43] | Inverse design | 625 nm | 66.66 | 0.48  0.32  0.25 | 1.07  1.7  2.32 | 16($\pm2$)  30($\pm2$)  44($\pm2$) | 16.34%  20.12%  23.48% |
| Our work | Topology-shape optimization | 632.8nm | 10 | 0.33 | 1.75 | 18.80 | 72.57% |

S6. Fabrication procedure

The metalens is composed of SiO_2_ as the substrate and TiO_2_ as the upper structure. First, a 600 nm thick TiO_2_ film could be coated on the substrate of SiO_2_ by the atomic layer deposition (ALD) technique. The photoresist is spin-coated and the electron beam lithography（EBL) etching technique could be used to obtain the desired shape of the photoresist structure. Then, the desired structure could be etched on the TiO_2_ layer using the inductively coupled plasma (ICP) etching technique. Finally, the top layer photoresist is removed to obtain the metalens. In order to measure the results closer to the theoretical simulation, the linewidth of the structure is set to 100 nm with a tolerance error of about 10 nm.
